# Metabolomics reveals immunomodulation as a possible mechanism for the antibiotic effect of *Persicaria capitata* (Buch.-Ham. ex D. Don) H.Gross

**DOI:** 10.1007/s11306-018-1388-y

**Published:** 2018-06-26

**Authors:** Pei Han, Yong Huang, Yumin Xie, Wu Yang, Wenying Xiang, Peter J. Hylands, Cristina Legido-Quigley

**Affiliations:** 1 Institute of Pharmaceutical Science, Faculty of Life Sciences & Medicine, King’s College London, London, SE1 9NH UK; 2 Provincial Key Laboratory of Pharmaceutics in Guizhou Province, School of Pharmacy, Guizhou Medical University, Guiyang, Guizhou China; 3 The Systems Medicine Group, Steno Diabetes Center, Gentofte, Denmark

**Keywords:** Immunomodulation, Metabolomics, *P. capitata*, Urinary tract infection

## Abstract

**Introduction:**

In spite of advances in antibiotics, urinary tract infection (UTI) is still among the most common reasons for antibiotic medication worldwide. *Persicaria capitata* (Buch.-Ham. ex D. Don) H.Gross (*P. capitata*) is a herbal medicine used by the Miao people in China to treat UTI. However studies of its mechanism are challenging, owing to the complexity of *P. capitata* with multiple constituents acting on multiple metabolic pathways.

**Objective:**

The objective of this study was to explore the working mechanism of *P. capitata* on urinary tract infection.

**Methods:**

*Relinqing*® granule, which is solely made from aqueous extracts of the whole *P. capitata* plant, was used in this study. Urine metabolomics based on gas chromatography-mass spectroscopy was employed to assess the metabolic changes caused by administration of *Relinqing®* granule in a UTI mouse model. Female specific-pathogen-free Kunming mice were divided into control group (mock infection, saline treatment), model group (*E.coli* infection, saline treatment), *Relinqing®* group (*E.coli* infection, *Relinqing®* granule treatment), ciprofloxacin group (*E.coli* infection, ciprofloxacin treatment), and sham-*Relinqing®* group (no surgery, *Relinqing®* granule treatment).

**Results:**

The results showed that after the treatments, urine levels of itaconic acid in *Relinqing®* group increased by 4.9 fold and 11.3 fold compared with model and ciprofloxacin groups respectively. Itaconic acid is an endogenous antibacterial metabolite produced by macrophages, which also functions as a checkpoint for metabolic reprogramming of macrophage.

**Conclusion:**

Our findings suggest that this herbal medicine can cure urinary tract infection through modulation of immune system.

**Electronic supplementary material:**

The online version of this article (10.1007/s11306-018-1388-y) contains supplementary material, which is available to authorized users.

## Introduction

Major progress in the treatment of infectious diseases has led to a noteworthy reduction in the morbidity and mortality associated with these illnesses (Alanis 2005; Davies and Davies 2010). However, progress has been hampered by the damage of antibiotics to gut microbiota and the emergence of antibiotic resistant strains due to the frequent consumption of antibiotics (Goossens et al. 2005). Nowadays, antibiotic resistance has become a major threat to the public health (Butler et al. 2006; Foxman 2010). It can lengthen the duration of hospital stays and increase patients’ risk, which in turn increases social health-care costs (Zhanel et al. 2006). In southern and central Europe, the prevalence of antibiotic resistance is reaching an alarming level (Goossens et al. 2005) and it is estimated that in the European Union, around 25,000 patients die from multidrug-resistant bacterial infection annually (Lim et al. 2016).

Urinary tract infection is the second most common reason for antibiotic prescription (Farrell et al. 2003). Currently antibiotic treatment is the recommended therapy for urinary tract infection and it usually starts before the results of urine culture are available (Farrell et al. 2003; Foxman 2010; Zhanel et al. 2006). Ciprofloxacin, ampicillin, and nitrofurantoin are amongst the most frequently used antibiotics (Foxman 2010). *Escherichia coli*, the main pathogen, and other pathogens have become resistant to these commonly used antibiotics. As a result of this resistance, the antibiotics become ineffective and infections persist, causing prolonged illness, disability and in some cases, death. This phenomenon highlights the importance of controlling the usage of antibiotics and the urgency of exploring alternative therapies for urinary tract infection (Arslan et al. 2005; Di Martino et al. 2006).

Traditional Chinese medicine (TCM) has gained more and more popularity across the world in treating and preventing diseases. *Relinqing*® granule is a Chinese patented medicine solely based on *Persicaria capitata* (Buch.-Ham. ex D. Don) H.Gross (*P. capitata*). It was approved by the State Food and Drug Administration of China and is the best-selling drug.(Liao et al. 2011; Zhenget al. 2014). *P. capitata* is a TCM that has long been used to treat various urologic disorders by the Miao people in China (Zhenget al. 2014). It has considerable antibacterial and anti-inflammatory activities whilst having little toxicity (Liao et al. 2011). Thus, it could be an alternative to antibiotics in the treatment of urinary tract infection. While previous studies have successfully quantified and identified the chemical components (Li et al. 2007; Liao et al. 2011, 2013; Zhang et al. 2013; Zhao et al. 2010), to our knowledge, the mechanistic understanding of its holistic activity is still unknown. The chemical components are listed in Online Resource 1. The major reason lies in that *P. capitata*, like other TCMs, applies a multi-targeted approach with multiple active components.

Metabolomics provides a new opportunity to address the holistic therapeutic mechanism of TCM. Using metabolomics Lin et al. elucidated the possible anti-hyperuricemia mechanism of luteolin and luteolin-4′-*O*-glucoside (Lin et al. 2018). Liang et al. studied the mechanism of *Shuanglong Formula* on myocardial infarction (Liang et al. 2010).

Therefore, in this study, the aim is to investigate the working mechanism of *P. capitata* in a systematic way with GC–MS metabolomics on the urinary tract infected mouse model by using *Relinqing*® granule. It is to find out whether *P. capitata* possesses a metabolic effect when compared with an antibiotic and ultimately whether this mechanism could be an alternative to the classic therapy for urinary tract infection.

## Materials and methods

### Bacterial strains and culture medium

*Escherichia coli* ATCC25922 bacteria were purchased from the National Centre for Medical Culture Collection (China). Mueller–Hinton Agar culture medium (batch number 20140924-00), Nutrient Agar culture medium (batch number: 20140304-01) were purchased from Hangzhou Microbial Regent Co., Ltd (China).

### Chemical reagents

LC–MS grade water, LC–MS grade acetonitrile were purchased from VWR international (UK). LC–MS grade methanol, *N,O-bis*-(trimethylsilyl)trifluoro-acetamide (BSTFA) with 1% trimethylchlorosilane (TMCS), *O*-methoxyamine-HCl (MOX), succinic-d4 acid (98% purity), itaconic acid (96%) were purchased from Sigma-Aldrich (UK). Ciprofloxacin (batch number: 1130026) was purchased from Guangzhou Bai Yunshan Pharmaceutical General Factory (China), *Relinqing®* granule (batch number: 141005) was purchased from Guizhou Wei Men Pharmaceutical Company.

### UTI model

All of the animal studies followed the guidelines of the Committee on the Care and Use of Laboratory Animals in China. Female specific-pathogen-free (SPF) Kunming mice (20 ± 2 g, 7–8 weeks) (certificate No. SCXK 2014-0011), obtained from *Changsha Tianqin* Biotechnology Co., Ltd (China), were maintained under standard laboratory conditions (24 ± 2 °C, 50–60% relative humidity, and 12 h light/dark cycle) for 1 week of adaptation. The UTI model generation process was the same with the method used in Han et al.’s work (Han et al. 2017). Briefly, polypropylene tube (inner diameter 0.28 mm, outer diameter 0.61 mm, Smith Medical Company, UK) carrying 0.05 mL of *E. coli* suspension (1 × 10^9^ cfu/mL) was placed into the mice bladder. The injection process took no less than 30 s. After infection with *E. coli*, the mice were deprived of water for 4 h and then were placed back on normal water and food consumption.

### Group information and urine sample collection

**Fig. 1:**
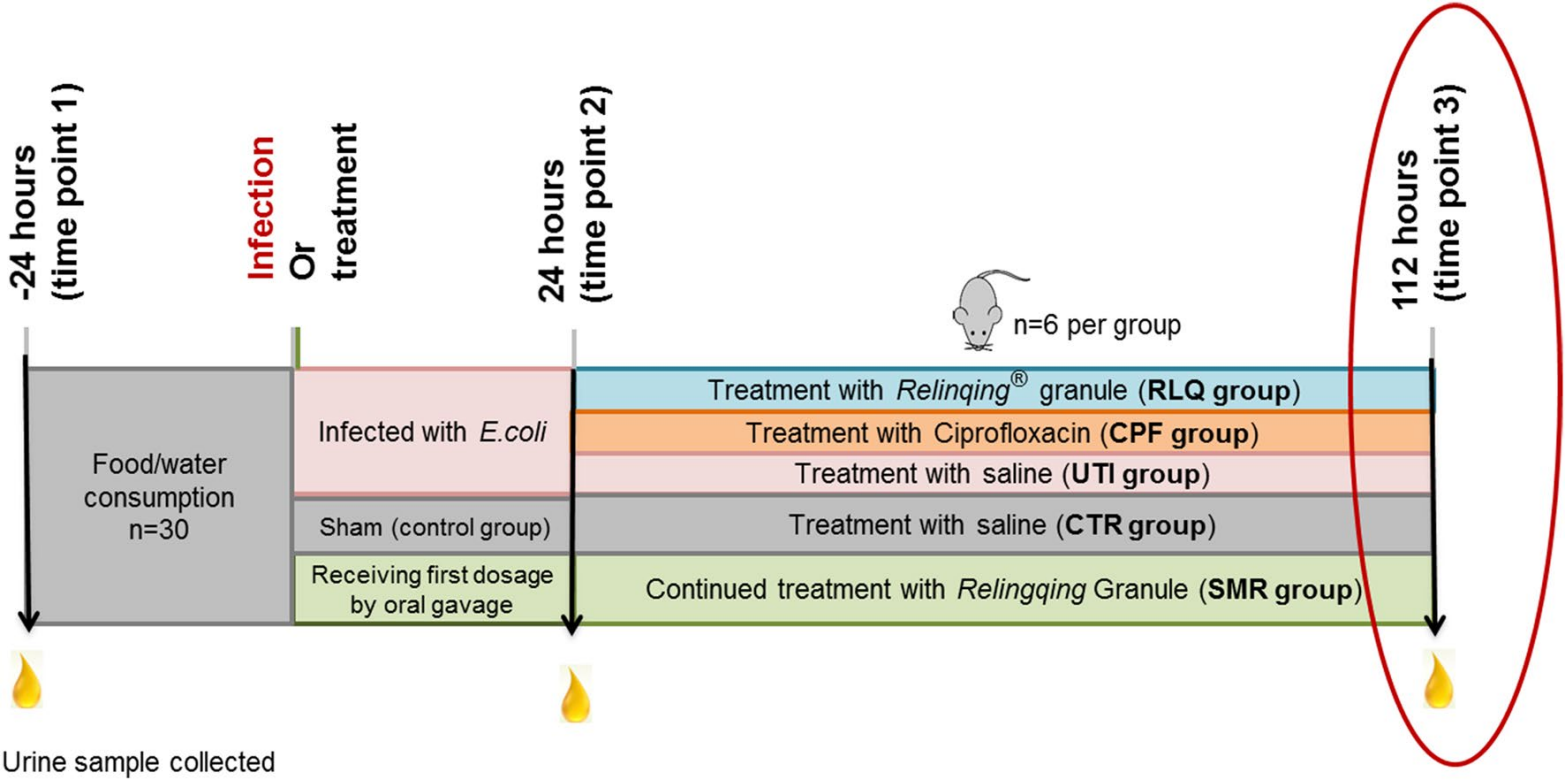
Graphic showing experiment design for the study of clinical mechanism of *P. capitata*. Metabolomics was performed at time point 3 (post-treatment). Figure adopted and modified from (Han et al. 2017)

Eighteen *E.coli*-infected mice were randomly divided into UTI (mice treated with saline after infection), CPF (mice treated with ciprofloxacin after infection) and RLQ (mice treated with *Relinqing®*) groups. Another 12 uninfected mice were split into two groups: CTR and SMR groups. The mice in CTR group were mock-infected at infection step (by the instillation of saline solution) while the mice in SMR group did not undergo surgery, but received the first dosage of *Relinqing®* granule at the same time point. CPF group were administered ciprofloxacin at 0.081 g/kg. RLQ and SMR groups were dosed with *Relinqing®* granule at 1.73 g/kg. All of the mice received the treatments twice a day for 3 days by oral gavage. Urine samples were collected 24 h before infection (time point 1), 24 h after infection (time point 2) and 16 h after three-day treatment (time point 3). All the urine samples (20 µL) were lyophilized and stored in − 80 °C until use. The group information and sample collection were shown in Fig. 1.

### Urine pH measurement

The pH value of urine samples at all time points in SMR group were measured with short range pH papers (accurate pH measurements at each 0.5 pH interval) before they were lyophilized for metabolomics analysis.

### Sample preparation and GC–MS analysis for metabolomics

The sample preparation and GC–MS analysis were performed as described in Han et al.’s work and is explained in detail in Online Resource 2 (Han et al. 2017). In brief, lyophilized urine samples were reconstituted in water with succinic-d4 acid as internal standard. All the samples underwent oximation and subsequently silylation. Then the derivatized samples were pipetted to amber vials with inserts for GC–MS analysis.

GC–MS analysis was carried out on a Shimadzu GC-2010 Plus gas chromatograph equipped with a GC–MS-QP2010 SE single quadruple mass spectrometer (Shimadzu, Kyoto, Japan). Samples were analyzed in a randomized order, with a pooled urine sample being used as quality control sample (QC sample) at regular intervals throughout the run.

### Data pre-processing and statistical analysis

Raw data were converted to .mzXML format in GC–MS Postrun Analysis (Shimadzu, Kyoto, Japan) before being processed by freely available “XCMS” package in R. The output table encompassing time-aligned features (retention time—mass to charge ratio pair), feature intensity and sample names were ready for normalization in R.

The normalized urine data were evaluated in SIMCA version 14 (MKS Umetrics AB, Sweden) for multivariate analysis. Before being subjected to orthogonal partial least squares-discriminant analysis (OPLS-DA) with corresponding S-plot analysis (feature selection criteria p[1] > 0.2, p(corr) > 0.6 and p[1] < − 0.1, p(corr) < − 0.8), data were logarithmically transformed (base10) and Pareto-scaled. Semi-quantification was performed by peak area of feature divided by peak area of internal standard and the data were presented as mean ± standard deviation (S.D.). All the statistic tests were performed in SPSS (IBM SPSS statistics, version 22). Graphs were prepared in SIMCA 14 and R (version 3.4.2) with package “ggplot2”.

### Putative metabolite identification

The features that were observed to contribute to the group separation were preliminarily identified by comparing fragmentation patterns of detected metabolites with the spectra in National Institute of Standards and Technology (NIST) library. The most important discriminating metabolic features were compared with pure standards to confirm the identities.

## Results and discussion

### Urine pH measurement

The pH of urine in SMR group was tested to see the effect of *Relinqing®* granule on urine pH. After dosing with *Relinqing®* granule, a significant drop (decreased by 1.09 fold, *p* < 0.05) of urine pH value between pre-dose and 1-day dose, pre-dose and 3-day dose was found, however there was no significant difference between 1-day dose and 3-day dose in terms of urine pH (shown in Online Resource 3).

Previously, Han et al. demonstrated that after *Relinqing®* treatment, urine bacteria number decreased significantly (decreased by 12.46 fold), indicating the effectiveness of *Relinqing®* granule (Han et al. 2017).

### Effects of *Relinqing*® granule based intervention on metabolite profiling

#### Data overview

A total of 2215 ionization features were initially extracted per chromatogram. A typical GC–MS chromatogram of a urine sample is illustrated in Online Resource 4a. A principal component analysis (PCA) modelling shows in yellow the extraction profile from a pooled urine sample, indicating good across-run reproducibility (Online Resource 4b).

#### Multivariate analysis of time point 3

In order to gain more insight into treatment effect on metabolic changes, four OPLS-DA models were built with the urine data at time point 3: (a) RLQ versus CTR, (b) RLQ versus UTI, (c) RLQ versus SMR, and (d) SMR versus CTR. The first three comparisons were made to examine the features related with the possible working mechanism whilst the last comparison was used to exclude any metabolic changes caused by *Relinqing®* treatment that might not be relevant to its mechanism of action. S-plot was used to generate a list of features of interest that were important for group separation. In total, 17 features with high p(corr) values were selected that contributed to group discrimination in each comparison (Online Resource 5). After checking the raw chromatograms, one was excluded as its concentration was too low and 10 were putatively identified. Among the 10 features, 4 were found to possess known biological functions and showed different levels among the groups. These were hippuric acid, *cis*-aconitic acid, itaconic acid, citric acid (similarity index > 90).

#### Hippuric acid

**Fig. 2:**
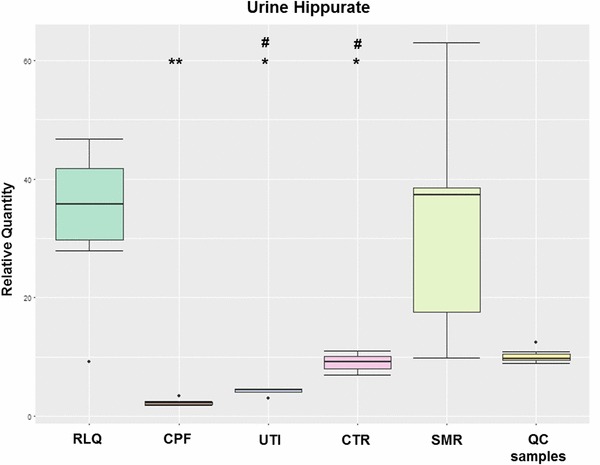
Box plot of urine hippurate levels in different groups after treatment (Mann–Whitney, *^,#^*q* < 0.05, **^,##^*q* < 0.001, *q* value is *p* value adjusted by Benjamini and Hochberg correction, * comparison made between RLQ group and the others; ^#^, comparison made between CPF and CTR, UTI groups). The peak area of hippurate in the raw data was normalized to the internal standard. The whiskers represent the highest and lowest values that are not outliers. Circles beyond the whiskers indicate the outliers. QC samples are the pooled urine samples showing analytical variation

Boxplot (Fig. 2) showed an elevation of urine hippurate after administration of *Relingqing®* granule in both RLQ and SMR groups compared with other groups. In the CPF group (mice treated with ciprofloxacin), the level of hippurate was the lowest and, compared to CTR group, it showed a 3.8 fold decrease.

Hippurate has been previously linked to the UTI prevention properties of cranberry as reports have shown an increased excretion of urine hippurate after consumption of cranberry products (Fellers et al. 1933; Moen 1962). Hippurate is known to be bacteriostatic, but as demonstrated by Bodel et al. hippurate could only exert bacteriostatic effect at pH 5.0 (Bodel et al. 1959; Raz et al. 2004). Considering urine pH in the present study, the pH did not decrease enough to support this mechanism.

Nevertheless, urine hippurate is also a gut microbial-mammalian co-metabolite that is closely related with gut microbiota activity and diet. The disturbance in urine hippurate level is often attributed to the perturbation of gut microbial activity (Holmes et al. 2008; Lees et al. 2013). Hippurate is primarily derived from aromatic acids and polyphenols via the action of gut microbiota (Phipps et al. 1998). From the present data, a significant rise was seen in RLQ and SMR groups. In RLQ group, the levels of hippurate increased by 3.65, 7.82, 13.87 and 0.99 compared to CTR, UTI,CPF and SMR. In SMR, the respective fold changes were 3.66, 7.89 and 13.97 (compared to CTR,UTI and CPF). Considering *Relingqing®* granule is the aqueous extraction of *P. capitata* which is rich in polyphenols (Liao et al. 2011), elevated urine hippurate levels in both RLQ and SMR groups compared to the other groups could be the indicator of normal function of the gut microbiota.

By contrast, the mice treated with ciprofloxacin showed reduced urine levels of hippurate compared to UTI and control groups. It is known that antibiotics could decrease the diversity, the number and evenness of the bacterial community in the gut, leading to a reduction in urine hippurate (Dethlefsen et al. 2008). In this case, this result suggests that *Relingqing®* granule would disturb less gut microbiota balance.

#### TCA cycle

**Fig. 3:**
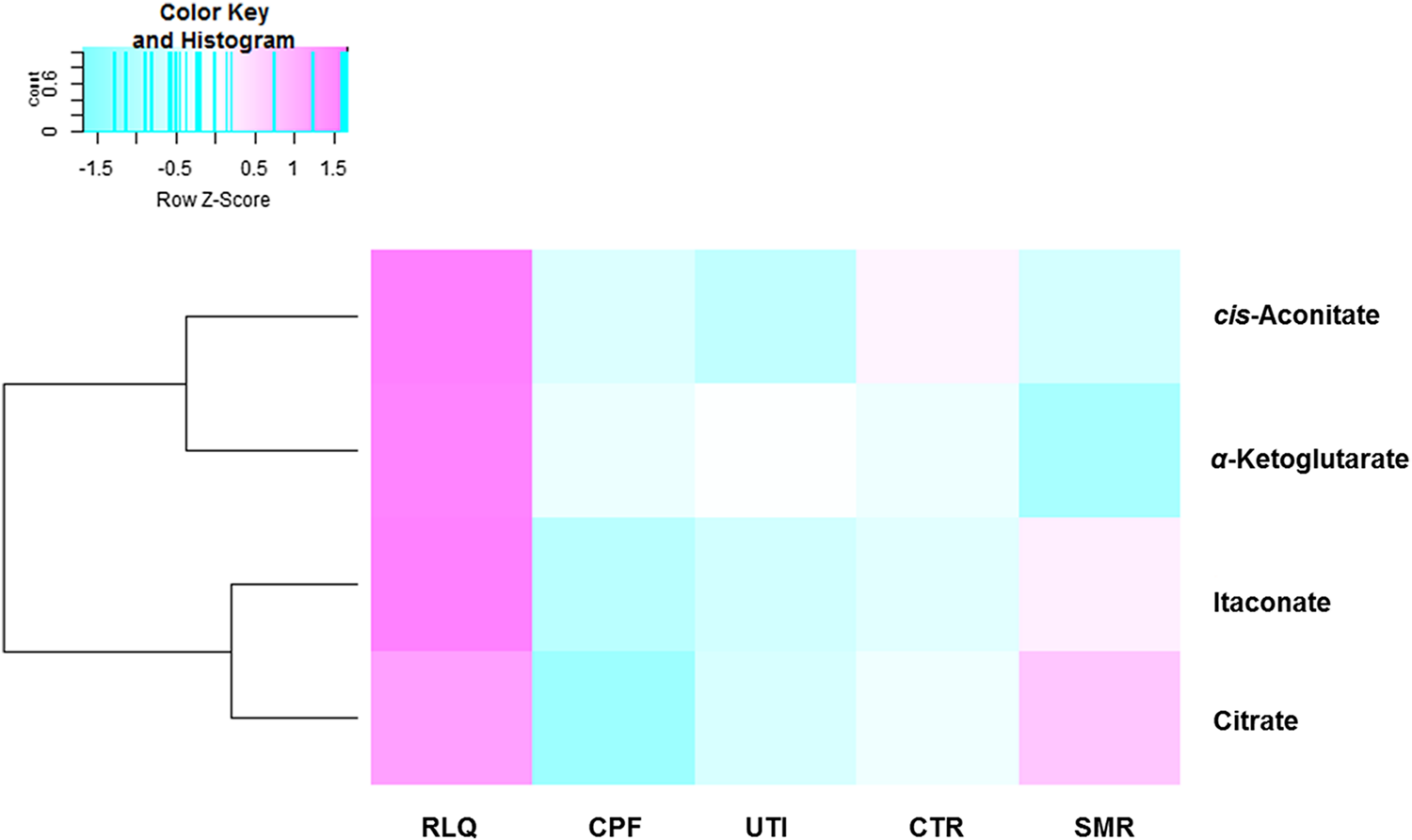
Heat-map showing the level of each metabolite in different group after three-day treatment (time point 3, n = 6 per group). Values are median quantities per group. Pink depicts increased quantity and blue means decreased quantity. (Color figure online)

Citric acid and *cis*-aconitic acid both belong to tricarboxylic acid (TCA) cycle. Itaconic acid is produced by *cis*-aconitic acid decarboxylation (Cordes et al. 2015). α-Ketoglutarate is the downstream product of *cis*-aconitic acid in the TCA cycle, so α-ketoglutarate level was also measured here. According to these data, all of these small metabolites presented a higher level in the RLQ group at time point 3 (see Fig. 3).

**Fig. 4:**
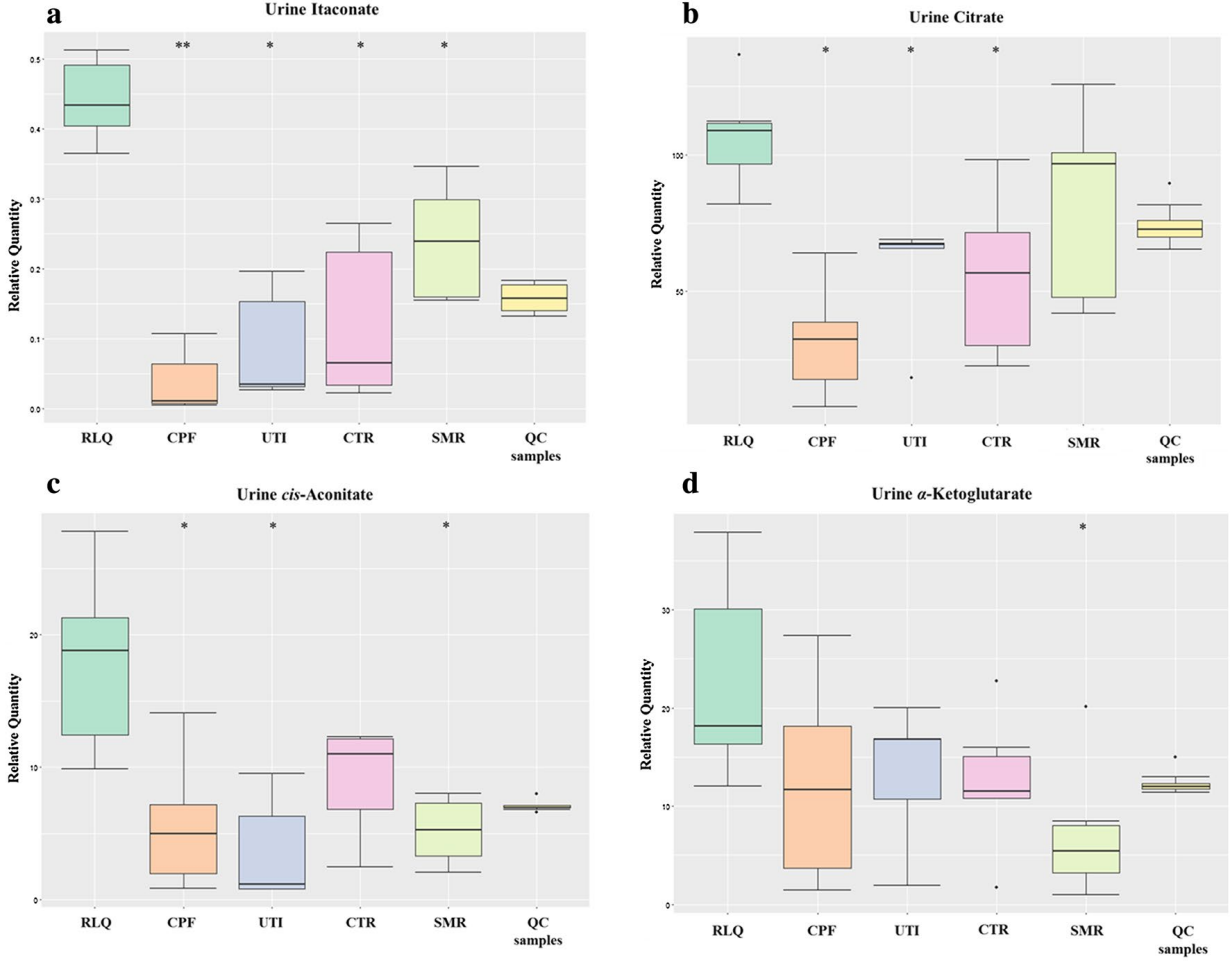
Box plots showing levels of **a** itaconate, **b** citrate, **c** *cis*-aconitate and **d** α-ketoglutarate among five groups at after treatment stage (Mann–Whitney *p* values are calculated from the comparison between RLQ and other groups, *q < 0.05, **q < 0.01; *q* value is *p* value adjusted by Benjamini and Hochberg correction.). QC samples are the pooled urine samples showing analytical variation

Among the four metabolites, itaconic acid displayed the greatest elevation in the RLQ group compared with the other three metabolites (Fig. 4a). The identity of itaconic acid was further confirmed by comparing against a pure reference standard (Online Resource 6). In the TCA cycle, α-ketoglutarate is the product from *cis*-aconitic acid. From Fig. 4, it can be observed that the level of *cis*-aconitic acid was much higher compared with the other four groups, while there was only a significant difference between RLQ and SMR groups in terms of α-ketoglutarate quantity. Thus, it can be suggested that *Relinqing®* granule propelled the conversion from *cis*-aconitic acid to itaconic acid, rather than α-ketoglutarate.

**Fig. 5:**
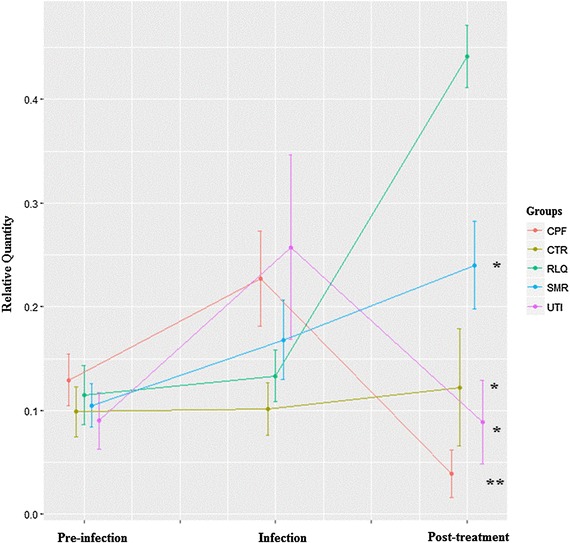
The change of urine itaconic acid from pre-infection to post-treatment (Mann–Whitney *p* values are calculated from the comparison between RLQ and other groups, *q < 0.05, **q < 0.01; *q* value is *p* value adjusted by Benjamini and Hochberg correction). CPF: mice treated with ciprofloxacin after infection, RLQ: mice treated with *Relinqing*® after infection, SMR: mice treated with *Relinqing*® without undergoing surgery, CTR: Mice treated with saline after mock-infection, UTI: mice treated with saline after infection

The change of itaconic acid was also monitored from pre- infection to post-treatment (results shown in Fig. 5). Before infection, all the mice showed a similar baseline level of itaconic acid. After the infection, there was a perturbation of itaconic acid levels. After the treatment, in the RLQ group, the level of itaconic acid increased greatly. It has an elevation of 2.07 fold, 3.61 fold, 4.95 fold and 11.31 fold respectively compared with SMR, CTR, UTI and CPF groups. In the SMR group, where the mice received the first dosage of *Relingqing®* granule at time point 2, the level of itaconic acid was also upregulated, although it was not as significant as RLQ group. Hence the upregulation of itaconic acid might be involved in the mechanism of action of this herbal medicine.

**Fig. 6:**
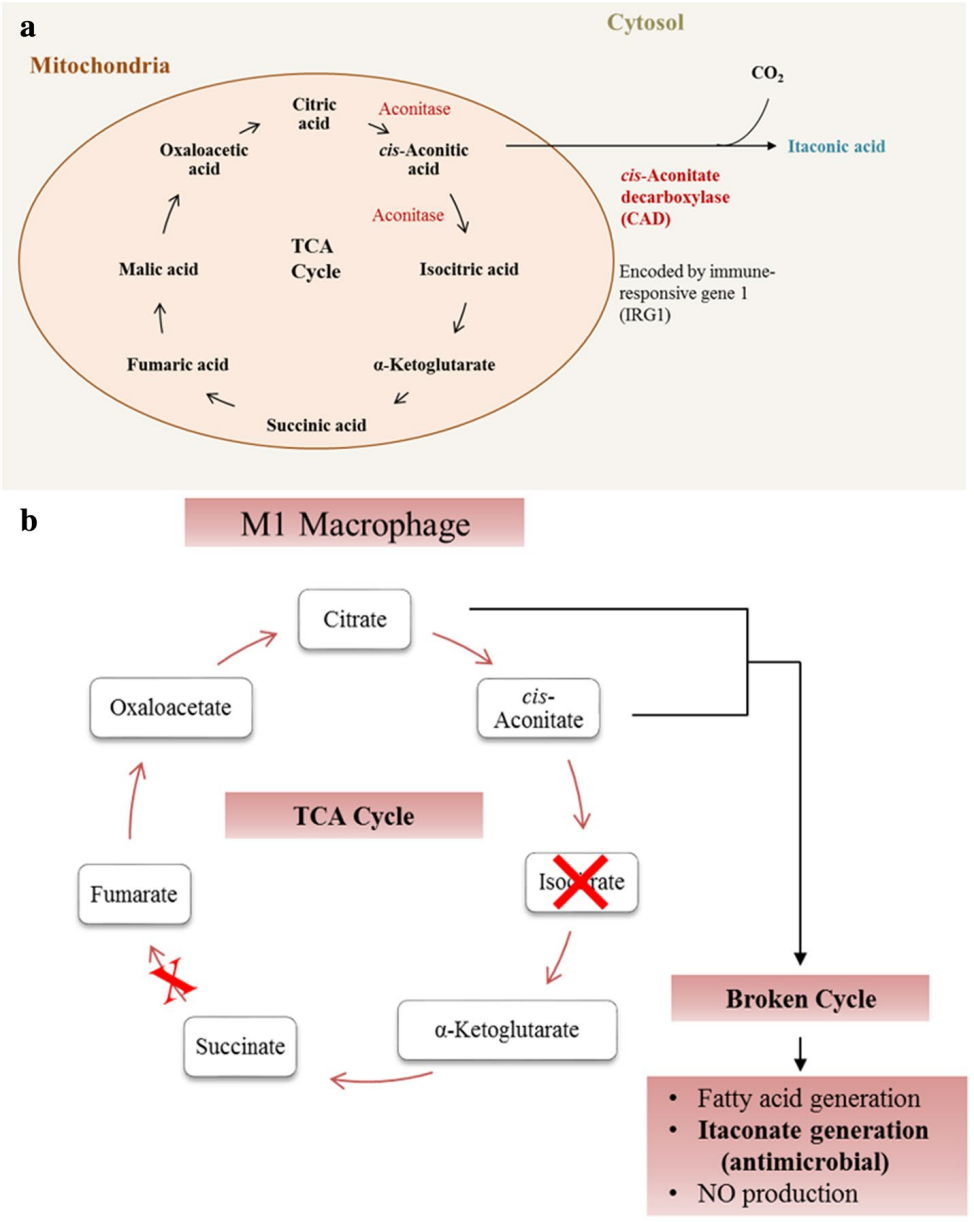
**a** Generation of itaconic acid in mammalian cell. **b** The TCA cycle in M1 macrophage. In M1 subtype, the TCA cycle was broken at isocitrate. *cis*-aconitate was unable to produce isocitrate, instead it was converted into itaconate which is an endogenous antimicrobial metabolite. Figure obtained and modified from (O’Neill et al. 2016)

Itaconic acid is an endogenous antibacterial compound and is converted from *cis*-aconitic acid (Fig. 6a) via the enzyme encoded by immune-responsive gene 1 (IRG1) (Cordes et al. 2015; O’Neill and Pearce 2016). It can inhibit the glyoxylate shunt which is indispensable for many pathogens such as *E.coli* to survive during the infection (Patel and McFadden 1978).

Itaconic acid is released by macrophages and its synthesis is distinctive to macrophage lineages (Strelko et al. 2011). Macrophages, part of myeloid cells, act as the first line of defence (Kang and Min 2012). This prodigious phagocyte is an essential modulator cell of innate immunity and also the principal element against infections (Schepetkin and Quinn 2006), since stimulating phagocytes is the key to immunity in host defence (Mosser and Edwards 2008).

Earlier studies have shown that activated macrophages generate large amounts of itaconic acid (Cheng et al. 2013; Strelko et al. 2011). The increased level of itaconic acid in RLQ group after treatment could indicate an enhanced phagocytic activity of macrophages in response to infection. But more importantly, this increase also implied a metabolic reprogramming in macrophage after *Relinqing*® granule treatment. Macrophage has two phenotypes. One is pro-inflammatory M1 phenotype, activated by lipopolysaccharides (LPS), the other is pro-reparatory M2 phenotype, triggered by IL-4 (O’Neill et al. 2016; O’Neill and Pearce 2016). These two macrophages adopt different metabolic pathways after activation. In M2 macrophage, there is still an intact TCA cycle. However, in M1 macrophage, the TCA cycle is broken at two places: at isocitrate site and after succinic acid (in Fig. 6b) and the resulted accumulation of citric acid was shown to serve as the direct chemical source for the production of itaconic acid (Jha et al. 2015; O’Neill et al. 2016). Thus, with the present data, it could be postulated that *P. capitata* was able to intensify this metabolic reprogramming in TCA cycle to reinforce the immune functions of macrophage to fight against infections. Therefore immunomodulation, which has been known as a very promising alternative to antibiotics (Bilitewski 2008), is very likely to be the reason why *P. capitata* is effective in UTI.

## Conclusions

In this study, a GC–MS based metabolomics method was applied to evaluate the working mechanism of *P. capitata* on an *E. coli* infected UTI mouse model and to find metabolites of its anti-bacterial effect. Hippuric acid, *cis*-aconitic acid, itaconic acid and citric acid were found to be increased in the RLQ groups.

The elevation of itaconic acid indicates that the mechanism of *P. capitata* involves enhanced macrophage activity and a possible alteration of TCA cycle in macrophages. This suggests that *P. capitata* may act as an immunomodulator to augment the host immune response.

## Electronic supplementary material

Below is the link to the electronic supplementary material.

Table S1 Major Components Presenting in *P. capitata*—Supplementary material 1 (PDF 83 KB)

Sample preparation and GC–MS analysis method—Supplementary material 2 (PDF 79 KB)

Table S2 Urine pH at three time points in SMR group—Supplementary material 3 (PDF 9 KB)

Fig. S1 Data overview of urine metabolomics analysis (a) Example GC–MS chromatogram of mouse urine sample. (b) PCA score plot of mouse urine samples. Yellow circles represent QC samples (n=11). The QCs cluster in the same area of the PCA score plot, indicating across-run reproducibility—Supplementary material 4 (TIF 330 KB)

Fig. S2 Venn diagram depicting the feature numbers obtained from different comparisons and the overlaps. The features were selected from S-plot to have values with p[1] > 0.2, p(corr) > 0.6 and p[1] < -0.1, p(corr) < -0.8. Comparison A compares RLQ and CTR; Comparison B compares RLQ and UTI; Comparison C compares RLQ and SMR—Supplementary material 5 (TIF 56 KB)

Fig. S3 Extracted ion chromatograms of a urine sample and an itaconic acid standard (a) Chromatogram and mass spectrum of itaconic acid reference standard. (b) Chromatogram and mass spectrum of the feature (m/z = 259) at retention time 10.22min obtained from a urine sample—Supplementary material 6 (TIF 255 KB)
