# Supplementary material for: Metabolomics reveals immunomodulation as a possible mechanism for the antibiotic effect of Persicaria capitata (Buch.-Ham. ex D. Don) H.Gross
Source: Metabolomics. 2018 Jun 26;14(7):91. doi: 10.1007/s11306-018-1388-y (PMC6019430; doi:10.1007/s11306-018-1388-y)
Supplement: Supplementary file 1 — Table S1 Major Components Presenting in P. capitata—Supplementary material 1 (PDF 83 KB) [file 11306_2018_1388_MOESM1_ESM.pdf]

Table S1 Major Components Presenting in *P.capitata*

Data adapted from the references cited in the main text

Components	Class	Molecular Weight
Gallic acid	Phenolic acids	170
Protocatechuic acid	Phenolic acids	154
Vanillic acid	Phenolic acids	168
Syringic acid	Phenolic acids	198
Catechin	Flavonoids	290
Rutin	Glycosides	610
Quercitrin	Glycosides	448
Quercetin	Flavonoids	302
Luteolin	Flavonoids	286
Kaempferol	Flavonoids	286
Schizandriside	Glycosides	492
Methyl gallate	Phenolic compounds	184
Ethyl gallate	Phenolic compounds	198
Ellagic acid	Tannins	302
Myricetrin	Glycosides	464
Que-3-*O*-Deoxyhex-4′-*O*-Hex	Glycosides	610
Myr-3-*O*-Hex	Glycosides	480

Que-3-*O*-Gala	Glycosides	464
Que-3-*O*-β-D-Glc	Glycosides	464
Que-3-*O*-Ara	Glycosides	434
Kam-3-*O*-Gala	Glycosides	448
Que-3-*O*-(2″-*O*-galloyl)-β-D-Glc	Glycosides	616
Que-3-*O*-(2″-*O*-protocatechuoyl)-Hex	Glycosides	600
Que-3-*O*-(3″-*O*-galloyl)-β-D-Glc	Glycosides	616
Ellagitannin	Tannins	992
Que-3-*O*-α-L-Rha	Glycosides	448
Bistoroside B	Glycosides	736
3, 3″-Di-*O*-methylellagic acid	Tannins	330
Que-3-*O*-(2″-*O*-protocatechuoyl)-L-Rha	Glycosides	584
Kam-3-*O*-Glc	Glycosides	449
Kam-4′-*O*-Rut	Glycosides	594
Kam-3-*O*-α-L-Rha	Glycosides	432
Que-3-*O*-Xyl	Glycosides	434

Que: quercetin, Kam: Kaempferol, Myr: myricetin, Rha: rhamnoside, Hex: hexoside, Glc: glucoside, Gala: galactoside, Xyl: xyloside, Ara: arabinoside, Deoxyhex: deoxyhexoside, Rut: rutinoside.
