# Supplementary material for: Metabolomics reveals immunomodulation as a possible mechanism for the antibiotic effect of Persicaria capitata (Buch.-Ham. ex D. Don) H.Gross
Source: Metabolomics. 2018 Jun 26;14(7):91. doi: 10.1007/s11306-018-1388-y (PMC6019430; doi:10.1007/s11306-018-1388-y)
Supplement: Supplementary file 2 — Sample preparation and GC–MS analysis method—Supplementary material 2 (PDF 79 KB) [file 11306_2018_1388_MOESM2_ESM.pdf]

# Methods

**Sample Preparation** 20µL of water was added into the lyophilized urine samples in microcentrifuge tubes and vortex-mixed for 2min. Then 60µL of methanol containing 20µg/mL of succinic-d4 acid as internal standard was added for protein precipitation. Samples were centrifuged at 10,000g for 10 mins at 4°C. A 20µL aliquot of the supernatant was transferred to a new auto-sampler vial. A pooled sample from all urine samples was used as quality control (QC) sample.

All the samples were dried under $N_2$. Then 20µL of MOX in pyridine (20mg/mL) was added to the residue of urine and incubated for 30min at 70 °C. Samples were dried again and then silylation was carried out with 30µL of BSTFA (1% TMS) for 1h at 70 °C. Then the derivatized samples were pipetted to amber HPLC vials with inserts for GC-MS analysis.

**GC-MS Analysis** GC-MS analysis instrumentation included a Shimadzu GC-2010 Plus gas chromatograph equipped with a GCMS-QP2010 SE single quadruple mass spectrometer (Shimadzu, Kyoto, Japan). Chromatographic separation was achieved by using a BP5MS capillary column. The temperature program consisted of 60°C at the start, maintained for 1 minute and then rose to 320°C at a rate of 10°C/min and held for 4minutes. Helium was the carrier gas with a flow rate set at 40cm/sec. The injection temperature, ion source temperature and interface temperature were set at 280°C, 200°C and 320°C, respectively. 0.5µL of derivatized urine sample was injected in the split mode with a split ratio of 1:60. Solvent cut time was 5 minutes. Mass spectra were scanned from m/z 50-600Da in SCAN mode with an electron impact ionization of 70eV.
