# Supplementary material for: Metabolomics reveals immunomodulation as a possible mechanism for the antibiotic effect of Persicaria capitata (Buch.-Ham. ex D. Don) H.Gross
Source: Metabolomics. 2018 Jun 26;14(7):91. doi: 10.1007/s11306-018-1388-y (PMC6019430; doi:10.1007/s11306-018-1388-y)
Supplement: Supplementary file 3 — Table S2 Urine pH at three time points in SMR group—Supplementary material 3 (PDF 9 KB) [file 11306_2018_1388_MOESM3_ESM.pdf]

Table S1 Urine pH at three time points in SMR group

	**Mouse 1**	**Mouse 2**	**Mouse 3**	**Mouse 4**	**Mouse 5**	**Mouse 6**	**Average**
**Pre-dose**	6.4	6.4	6.7	5.8	6.4	6.4	6.35
**1-day dose**	5.8	5.8	5.4	5.8	5.8	6.2	5.8[*a]
**3-day dose**	5.8	5.8	6.7	5.8	5.8	5.8	5.95[*a]

[a] Compared to pre-dose. *p*-value was obtained from Wilcoxon signed-rank test, * $p < 0.05$
