# Supplementary figures and images for: Metabolomics reveals immunomodulation as a possible mechanism for the antibiotic effect of Persicaria capitata (Buch.-Ham. ex D. Don) H.Gross
Source: Metabolomics. 2018 Jun 26;14(7):91. doi: 10.1007/s11306-018-1388-y (PMC6019430; doi:10.1007/s11306-018-1388-y)

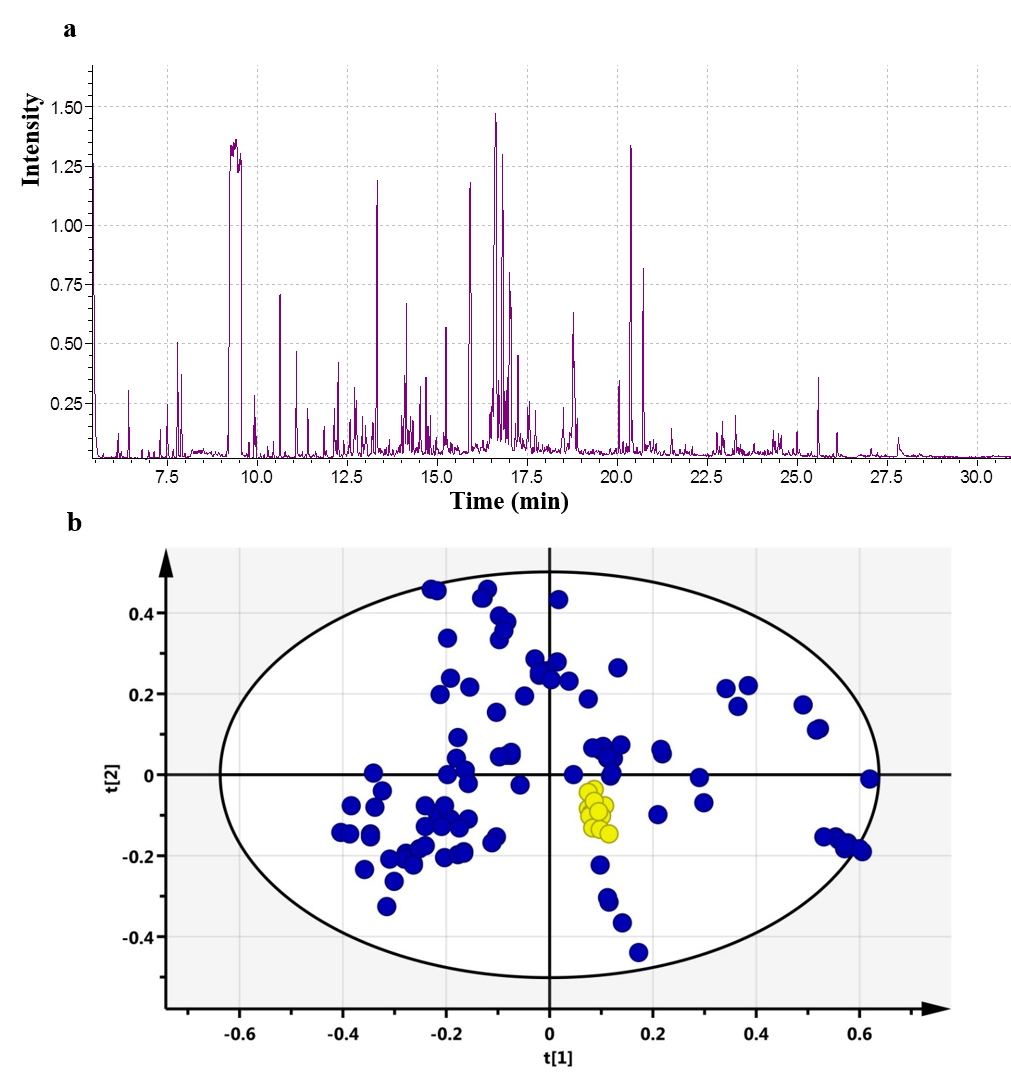

Supplement: Supplementary file 4 — Fig. S1 Data overview of urine metabolomics analysis (a) Example GC–MS chromatogram of mouse urine sample. (b) PCA score plot of mouse urine samples. Yellow circles represent QC samples (n=11). The QCs cluster in the same area of the PCA score plot, indicating across-run reproducibility—Supplementary material 4 (TIF 330 KB) [file 11306_2018_1388_MOESM4_ESM.tif]

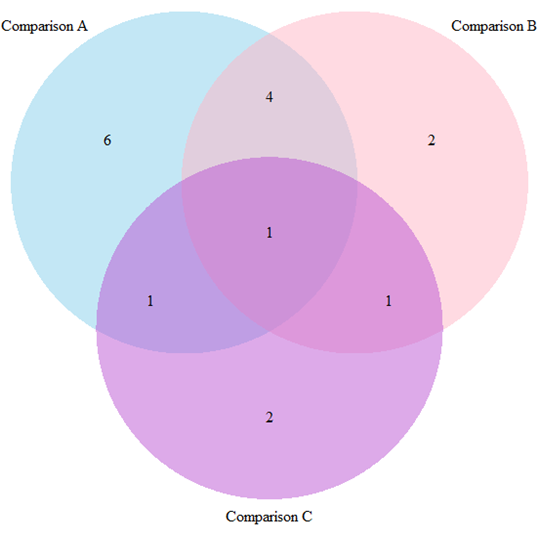

Supplement: Supplementary file 5 — Fig. S2 Venn diagram depicting the feature numbers obtained from different comparisons and the overlaps. The features were selected from S-plot to have values with p[1] > 0.2, p(corr) > 0.6 and p[1] < -0.1, p(corr) < -0.8. Comparison A compares RLQ and CTR; Comparison B compares RLQ and UTI; Comparison C compares RLQ and SMR—Supplementary material 5 (TIF 56 KB) [file 11306_2018_1388_MOESM5_ESM.tif]

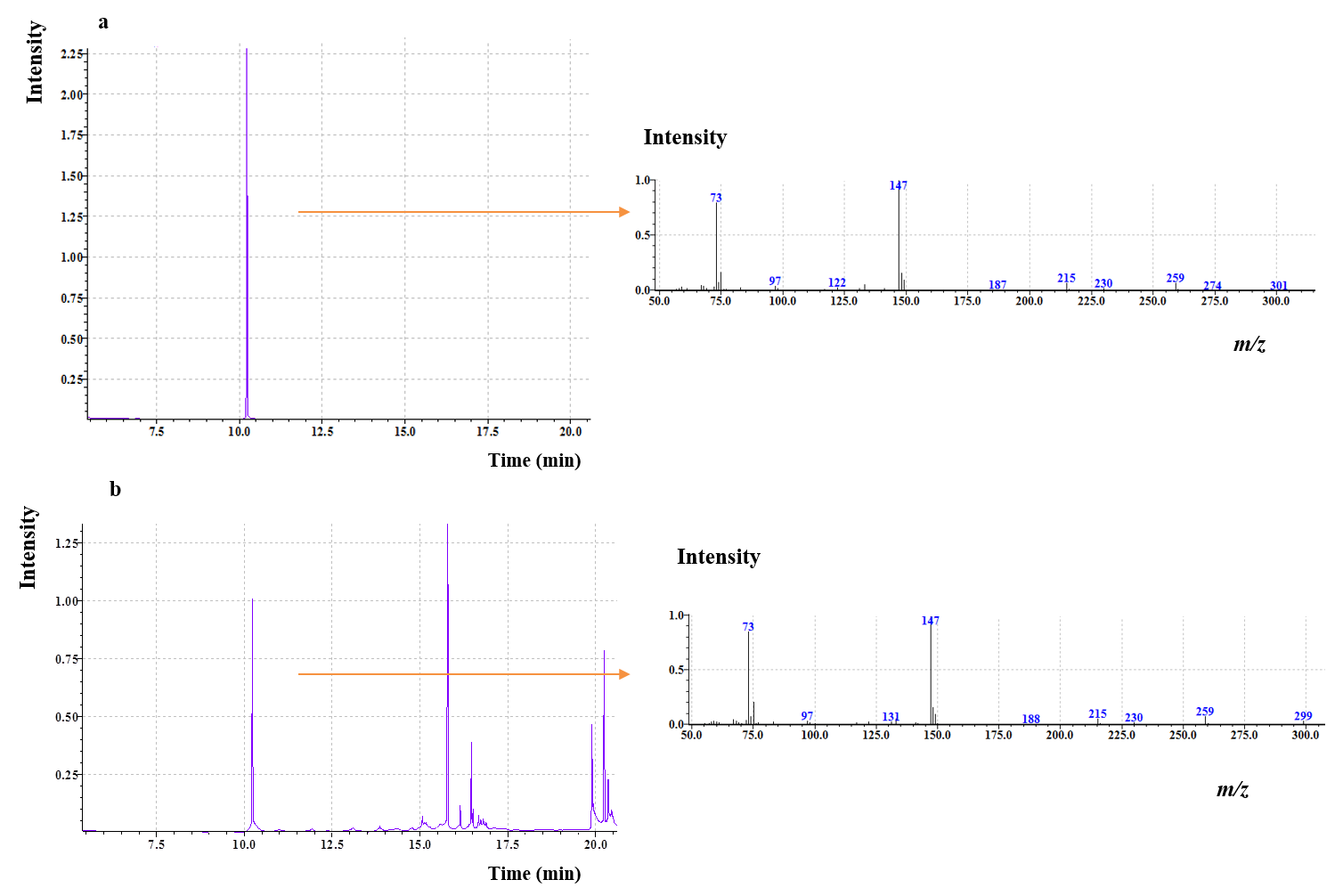

Supplement: Supplementary file 6 — Fig. S3 Extracted ion chromatograms of a urine sample and an itaconic acid standard (a) Chromatogram and mass spectrum of itaconic acid reference standard. (b) Chromatogram and mass spectrum of the feature (m/z = 259) at retention time 10.22min obtained from a urine sample—Supplementary material 6 (TIF 255 KB) [file 11306_2018_1388_MOESM6_ESM.tif]
